# Comparison of Diplodia Tip Blight Pathogens in Spanish and North American Pine Ecosystems

**DOI:** 10.3390/microorganisms9122565

**Published:** 2021-12-11

**Authors:** Ana Aragonés, Tania Manzanos, Glen Stanosz, Isabel A. Munck, Rosa Raposo, Margarita Elvira-Recuenco, Mónica Berbegal, Nebai Mesanza, Denise R. Smith, Michael Simmons, Stephen Wyka, Eugenia Iturritxa

**Affiliations:** 1 Neiker-BRTA, Instituto Vasco de Investigación y Desarrollo Agrario, Granja Modelo s/n, Antigua Carretera Nacional 1, Km. 355, 01192 Arkaute, Spain; aaragones@neiker.eus (A.A.); tmanzanos1001@gmail.com (T.M.); nmesanza@neiker.eus (N.M.); 2 Department of Forest and Wildlife Ecology, University of Wisconsin-Madison, Madison, WI 53706, USA; gstanosz@wisc.edu (G.S.); drsmith4@wisc.edu (D.R.S.); 3 Northeastern Area State and Private Forestry, USA Department of Agriculture Forest Service, Durham, NH 03824, USA; Isabel.Munck@usda.gov; 4 Intituto de Investigación Forestal_ Instituto Nacional de Investigación y Tecnología Agraria (CIFOR, INIA), Carretera La Coruña Km 7.5, 28040 Madrid, Spain; raposo@inia.es (R.R.); elvira@inia.es (M.E.-R.); 5 Sustainable Forest Management Research Institute, University of Valladolid-INIA, Avenida Madrid 44, 34004 Palencia, Spain; 6 Instituto Agroforestal Mediterráneo, Universitat Politècnica de València, Camino de Vera S/N, 46022 Valencia, Spain; mobermar@etsia.upv.es; 7 Department of Natural Resources and the Environment, University of New Hampshire, Durham, NH 03824, USA; Michael.Simmons@unh.edu; 8 LifeMine Therapeutics, Cambridge, MA 02140, USA; stephenwyka@gmail.com

**Keywords:** *Sphaeropsis sapinea*, Diplodia tip blight, dieback, SSRs, *Diplodia scrobiculata*

## Abstract

Diplodia tip blight is the most ubiquitous and abundant disease in Spanish *Pinus radiata* plantations. The economic losses in forest stands can be very severe because of its abundance in cones and seeds together with the low genetic diversity of the host. *Pinus resinosa* is not genetically diverse in North America either, and Diplodia shoot blight is a common disease. Disease control may require management designs to be adapted for each region. The genetic diversity of the pathogen could be an indicator of its virulence and spreading capacity. Our objective was to understand the diversity of *Diplodia* spp. in Spanish plantations and to compare it with the structure of American populations to collaborate in future management guidelines. Genotypic diversity was investigated using microsatellite markers. Eight loci (SS9–SS16) were polymorphic for the 322 isolates genotyped. The results indicate that *Diplodia sapinea* is the most frequent *Diplodia* species present in plantations of the north of Spain and has high genetic diversity. The higher genetic diversity recorded in Spain in comparison to previous studies could be influenced by the intensity of the sampling and the evidence about the remarkable influence of the sample type.

## 1. Introduction

*Pinus radiata* D. Don. is the most widespread exotic forest species susceptible to the fungal pathogen *Diplodia sapinea* (Fr.) Fuckel (syn. *Diplodia pinea* (Desm.) Kickx., *Sphaeropsis sapinea* (Fr.:Fr./Dyko and Sutton)) in Spain. The first record of the introduction of *P. radiata* D. Don in Spain is its presence in a garden in Lekeitio (Bizkaia) in the mid-19th century [1]. It was considered an appropriate candidate for forestry in Spain based on previous acclimatization studies [2]. The first plantations of this tree species in Spain were established at the end of the 19th century in the Basque Country. A policy of reforestation of public lands [3] led to an increase in the area covered by plantations, which reached over 160,000 ha by the 1970s [3,4,5]. Due to the environmental requirements of *P. radiata*, cold sensitivity and high humidity, its distribution within the northern Iberian Peninsula is limited mainly to the Cantabrian coast, which has an Atlantic climate, where this species is an important feature of the landscape [4,6]. It is difficult to determine the origin of *P. radiata* germplasm at any particular location in Spain [3], *P. radiata* seeds were obtained from collections performed during the first thinnings of the Basque Country pine forests and from providers located in New Zealand, the USA, Chile, France and Denmark. However, a study of the population of *P. radiata* growing in Spain showed low genetic diversity [7]. The local landrace and the three Californian natural provenances (Año Nuevo, Monterey and Cambria) have been compared using genetic diversity analysis by molecular markers (RAPDs), growth and characteristics morphological and survival. The local population was found to be most similar to the Año Nuevo provenance. The Año Nuevo and the local landrace showed the lowest mortality (2.1 and 8.1%, respectively). Mortality was greater for Monterey provenance (29.3%) and particularly high for the Cambrian provenance (52.6%). The differential genotype adaptation to local conditions of northern Spain and survival there may explain in part the detected low genetic diversity.

Diplodia tip blight caused by *D. sapinea* is the most ubiquitous and abundant disease in Spanish *P. radiata* plantations, which are monocultures and susceptible to various diseases. In a field survey performed in 2009 in the Basque Country [8], the incidence of Diplodia tip blight in surveyed plots was 100%, compared to 17% for pitch canker disease caused by *Fusarium subglutinans* f. sp. *pini.* The severe economic losses in forest stands of *P. radiata* can be attributed to both the low genetic diversity of the host [7,9] and abundance of the pathogen in cones and seeds [8,10,11,12] which are the major sources of inoculum in the area [13].

Similar to *P. radiata* in Spain, *P. resinosa* Ait. is one of the least genetically diverse conifer species in North America [14]. The native range of *P. resinosa* is a narrow latitudinal band running east–west across southeastern Canada and the northeastern USA. This range extends north to Maine (USA), southern Quebec (Canada), New Brunswick (Canada), and Nova Scotia (Canada), west to central Ontario (Canada), and south to Minnesota (USA), Wisconsin (USA), Michigan (USA), northern Pennsylvania (USA), northern New Jersey (USA), Connecticut (USA), and western Massachusetts (USA) [15]. In addition, separated patches of endemic *P. resinosa* also occur in Newfoundland (Canada), northern Illinois (USA), and eastern West Virginia (USA) [15]. Red pine is naturally found in pure stands or more commonly in mixtures with *Pinus strobus* (eastern white pine) or *Pinus banksiana* (jack pine) on well-drained sandy soils [15].

Red pine was widely planted in the 1930s to 1960s to stabilize abandoned agricultural lands [15]. Today, red pine is one of the most commonly planted trees in the northern USA and Canada [15]. Diplodia shoot blight, collar rot and canker are common diseases in these red pine plantations [16,17,18,19,20,21,22]. The persistence of *D. sapinea* in seed orchards and forest nurseries may have contributed to the widespread dissemination of this pathogen [13,23,24,25]. *Diplodia sapinea* was considered a variable organism, both in morphological and virulence of different strains but a recent study confirms that the European *D. sapinea* population is homogeneous and little differentiated except for subpopulations from Italy and Georgia [26]. Historically, three morphotypes were differentiated [27,28,29]. In 2003, de Wet et al. [30], however, proposed the separation of morphotypes into species. Based on comparison of multiple genes and microsatellite markers, they concluded that strains of morphotypes A and C corresponded to *D. sapinea*. For the morphotype B strains, they proposed a new taxon called *Diplodia scrobiculata* J. de Wet, Slippers & M. J. Wingf. Both species have been detected in Spain on *P. radiata* [31] and North America on *P. resinosa* [32,33].

Control of *D. sapinea* is complicated because it is capable of surviving on needles, branches, shoots, wood and pine cones for long periods [20,23,34,35,36,37]. It is commonly isolated from seedlings, needles, cones, branches, seed scales, seeds and pits of cones and mature wood [25,38,39,40,41,42,43]. Furthermore, *D. sapinea* can persist asymptomatically as a latent pathogen [10,11]. Only after trees experience a stress event such as drought, physical damage or hail may the characteristic Diplodia tip blight symptoms develop [44,45,46,47]. In addition, its genetic diversity could be an indicator of its potential virulence and capacity to spread.

The frequent importation of *P. radiata* seeds in Spain from different suppliers of uncertain origin and the negative impact of these diseases on the productivity of our forest stands led us to carry out this study. Our objective was to understand the diversity of *Diplodia* spp. in Spanish *P. radiata* plantations and to compare the structure of American *D. sapinea* populations with populations obtained from *P. radiata* in northern Spain. Most of the North American *D. sapinea* isolates used in this study came from *P. resinosa* plantations, which are similar to *P. radiata* plantations in the low genetic variation of the hosts and potential spread of the pathogen via nursery stock. Improved knowledge about the genetic diversity and mode of reproduction in the Basque Country *D. sapinea* populations might help to design specific management options at a local scale. In addition, the potential influence of the sampling strategy (density and sample type) will be discussed.

## 2. Materials and Methods

### 2.1. Fungal Collection and Isolation

Symptomatic (dieback) *P. radiata* trees were sampled in the major pine-growing regions of the Basque Country during spring and summer of 2016–2020. Plantations located in Laukiniz (P1), Sollano (P2), Hernani (P3), Luyando (P4) and Oiartzun (P5) were intensively sampled. Otherwise, only one strain of *Diplodia* spp. per plot was isolated. Fragments of cone scales bearing pycnidia were soaked in 30% commercial bleach (1.6% sodium hypochlorite) for 1 min and rinsed with sterile water. A single pycnidium from the cone surface was transferred to water agar medium (Panreac, Barcelona, Spain), and a single conidium was selected to initiate a monosporic culture. Single conidial isolates were grown on potato dextrose agar (Panreac, Barcelona, Spain) in petri plates in darkness at 20 ± 3 °C for 4 to 6 days. Fungal species were initially identified by colony and conidium morphology [30,48]. *Diplodia* species were confirmed by molecular methods. All isolates were maintained in the Culture Collection of the Forestry Department, Neiker, BRTA Granja Modelo Arkaute, Vitoria-Gasteiz, Spain.

Roots were carefully washed under tap water to remove any adhered soil particles. For surface disinfestation, roots were dipped into 70% EtOH for 1 min, submerged in a 30% commercial bleach with Tween 20 (1 drop/100 mL) solution for 15 min and rinsed twice in sterile distilled water. The thinnest roots (less than 1 mm diameter) were immersed in the same commercial bleach solution but for 10 min instead of 15 min. These thin surface disinfested roots were aseptically transferred to sterilized filter paper and when dried, transversally cut into 5 mm long segments and placed on PDA petri dishes. The thickest roots were first longitudinally divided into two pieces and then cut into 5 mm long pieces.

In addition, branches, needles, pieces of wood from cankers and cores were sampled. Cores were collected from tree trunks with a Pressler’s 5-mm-diameter increment borer at 130 cm height [49]. Needles, fragments of branches and wood were separately collected in paper bags, and cores were introduced into sterilized tubes. All the tools in contact with the samples were disinfected before and after sampling with 70% EtOH. All the samples were labelled and stored at 4 °C. In the laboratory, the samples were immersed for 2 min in a sodium hypochlorite solution (1% active chlorine) and rinsed with sterile water. Thin disks cut from whole cross sections of the cores and branches were placed on potato dextrose agar (Panreac, Bareclona, Spain) and cultivated under the same conditions as the conidial cultures.

Three to six petri dishes per sample were used. Dishes were incubated in darkness at 25 °C and evaluated every 3 days. Putative colonies of *D. sapinea* were transferred to potato dextrose agar (PDA) dishes, which were incubated for 7 days at 25 °C, and mycelial growth characteristics were observed. Isolates were then grown on 2% water agar with sterilized pine needles at 25 °C under near-ultraviolet light (near-UV light) to induce sporulation.

North American isolates were obtained from an extensive collection at the University of Wisconsin-Madison and *P. resinosa* cones collected in New England, USA. Isolates U1–U50 were obtained as part of a study evaluating the factors and effects associated with widespread red pine mortality. *Pinus resinosa* branch and cone samples were obtained from asymptomatic trees and trees expressing crown dieback symptoms associated with *Matsucoccus matumurae* Kuwana (pine bast scale) infestation within both plantations and natural stands, the latter of which were located in Hancock County, Maine..

*Pinus resinosa* cones from New England were sampled using methods described previously [50]. Cones were bagged, placed on ice in a cooler for transportation, and stored in a freezer until processed in the laboratory. Conidia were extracted from each cone, and *Diplodia* species were identified by morphological and molecular methods described below. Isolates were sent to Neiker’s lab to implement the molecular work. 

### 2.2. Species Identification

Morphological and molecular methods were used to identify isolates. Conidial shape, color, presence of septa, width and length were observed, as well as mycelial growth. Mycelium grown on medium in petri dishes was scraped off and collected in a 2 mL tube with five sterile tungsten carbide beads (300 μM diameter). The fungal material was disrupted using a Qiagen-Retsch MM300 Tissuelyser (Qiagen, Hilden, Germany) at a speed of 30 m/s for 3 min at room temperature. In all cases, fungal DNA was extracted from 200 mg pure monosporic cultures using a DNA Plant Mini Kit (Analytik Jena AG, Life Science). Extractions were performed following the manufacturer’s instructions. DC-PCR with species-specific primers was used to differentiate *D. sapinea* DpF (5′-CTTATATATCAAACTATGCTTTG-TA-3′) and *D. scrobiculata* DsF (5′-CTTATATATCAAACTAATGTTTG-CA-3′); a Botryosphaeria-specific primer was used as the reverse primer BotR (5′-GCTTACACTTTCATTTATAGACC-3′) [18] and was used for the identification of species. PCR amplification was performed in a total volume of 25 μL containing 1 x reaction buffer, 2 mM MgCl_2_, 0.25 mM dNTPs, 0,8 μM of each specific primers, 40 ng of DNA and 1.25 U Platinum Taq polymerase (Roche Diagnostic GmbH, Mannheim, Germany). The cycling profile was as follows: denaturation at 94 °C for 60 s, followed by 35 cycles at 94 °C for 30 s, 67 °C for 30 s, and 72 °C for 30 s, and a final extension at 70 °C for 5 min. Fragment sizes were verified on 0.7% agarose gels in Tris-boric acid-EDTA buffer (TBE) with DNA loading buffer, 5× DNA (Bioline Merdidian Bioscience, London, UK).

### 2.3. PCR Amplification of SSR Loci and Data Analysis

Ten microsatellite loci, SS1-5-9-10-11, previously described by Burgess et al. [51], and SS12-14-15-16, described by Bihon et al. [52], were amplified for 322 *D. sapinea* isolates (Table 1). Positive controls with known DNA and negative controls without DNA were included. All SSR-PCR products were multiplexed and run in a single lane. SSR-PCR was conducted with a PCR mixture containing 1× QIAGEN^®^ Multiplex PCR kit, 0.15 μM (each) primer, 15 ng of DNA template and water to a final volume of 13 µL. The reactions were carried out in a thermocycler (Eppendorf, Hamburg, Germany) programmed for an initial denaturation of 1 min at 95 °C, followed by 2 min at 94 °C, 15 cycles of 30 s at 58 °C, 45 s at 60 °C and 1 min at 72 °C, and 20 cycles of 55 °C. Dilution of 1:50 for the thermocycler products was conducted before multiplex analysis to avoid detection error. The forward primers were labelled with a phosphoramidite fluorescent dye indicated as FAM, NED, PET and VIC.

One µL of these multiplexed PCR products was separated on an ABI Prism 3130 Genetic Analyser (Applied Biosystems, Foster, CA, USA). The amplicon peaks were determined based on the four fluorescent dyes used and the sizes of the DNA fragments. The mobility of SSR products was compared to those of internal size standards (LIZ-500), and allele sizes were estimated by GeneMapper 4.0 computer software (Applied Biosystems, Foster, CA, USA). A reference sample was run on every gel to ensure reproducibility. 

For each population defined by country of origin (the Basque Country in Spain and USA), nursery location within the Basque Country and sample type, the total number of alleles at each SSR locus was estimated. A multilocus genotype (MLG) was constructed for each isolate by combining data for single SSR alleles, and the expected multilocus genotype (eMLG) based on rarefaction was calculated using the R package poppr V.2.3.0 [53,54]. Given the clonality observed analyses were conducted for the clone-corrected dataset, with only one isolate of each MLG considered. Stoddart and Taylor’s diversity index (G) [55] and evenness index E5 [56] were calculated using the same R package.

The standardized index of association (rbarD) as an estimate of linkage disequilibrium was calculated to investigate the mode of reproduction [54,57]. The expectation of rbarD for a randomly mating population is zero, and significant deviation from this value would suggest clonal reproduction. Significance was tested based on 1000 permutations and conducted in the R package poppr using the clone-corrected data [54].

The standardized measure of genetic differentiation G´st described by Hedrick [58] was calculated to estimate subdivision among populations. This index ranges from 0 to 1, independent of the extent of population genetic variations and locus mutation rates [58]. Pairwise G´st values within the clone-corrected data were calculated using the R packages strata G V.1.0.5 [59] and mmod V.1.3.3 [60]. Statistical significance was calculated based on 1000 permutations.

Discriminant analysis of principal components (DAPC) was performed to infer clusters of populations without considering previous geographic/nursery location/isolation tissue-based assignment criteria [61]. DAPC was conducted with the R package adegenet V. 2.0.1 [62] using the Bayesian information criterion (BIC) to infer the optimal number of groups. Important advantages of DAPC are that it maximizes variation between the groups, minimizes the within-group genetic variability and does not require assumptions regarding evolutionary models [61].

To assess the relationships among MLGs, minimum spanning networks (MSNs) were constructed from the clone-corrected dataset. Bruvos´s genetic distance matrix and MSNs were generated using the R package poppr V.2.3.0 [53,54]. The genetic distance described by Bruvo et al. [63] takes the SSR repeat number into account, with a distance of 0.1 equivalent to one mutational step (one repeat).

### 2.4. DNA Sequencing and Phylogenetic Analysis

Based on different MLGs, 47 *D. sapinea* isolates were selected. The internal transcribed spacer (ITS) region was amplified using the primers ITS1 and ITS4 [64], and translation elongation Factor 1-α (TEF1-α) was amplified using the primers EF1-728F and EF1-986R [65]. PCRs for each region contained 20 ng DNA, 3 μL 10× PCR Complete KCl reaction buffer (IBIAN Technologies) containing 15 mM MgCl2, 200 nM of each primer, 200 μM of each dNTP and 1 U IBIAN-Taq DNA polymerase (IBIAN Technologies, Zaragoza, Spain). The PCR profile for the ITS region was as follows: 94 °C for 10 min, 35 cycles at 94 °C for 30 s, 58 °C for 45 s, 72 °C for 60 s, and 72 °C for 10 min. For the TEF1-α region, the same PCR conditions were used, but the annealing temperature was set at 52 °C. PCR products were sequenced by Macrogen (Seoul, South Korea).

Sequence data were edited using FinchTV software version 1.4.0 (https://finchtv.software.informer.com/1.4/, accessed on 8 January 2021) and aligned, and a phylogenetic tree was constructed from the aligned sequences with MEGA X software version 10.0.4 (https://www.megasoftware.net/, accessed on 3 October 2021).

## 3. Results

### 3.1. Species Identification

The presence of *Diplodia scrobiculata* was detected only in a single tree, from a wood core sample, of all the 253 analyzed trees in the Basque Country. *Diplodia scrobiculata* detection is considered something exceptional in this region where both *Diplodia* species co-occurred in the same tree. Only *D. sapinea* was isolated from red pine cones collected in New England. Table 1 shows the strains identified as *D.sapinea.*

### 3.2. PCR Amplification of SSR Loci and Data Analysis

**Figure 1:**
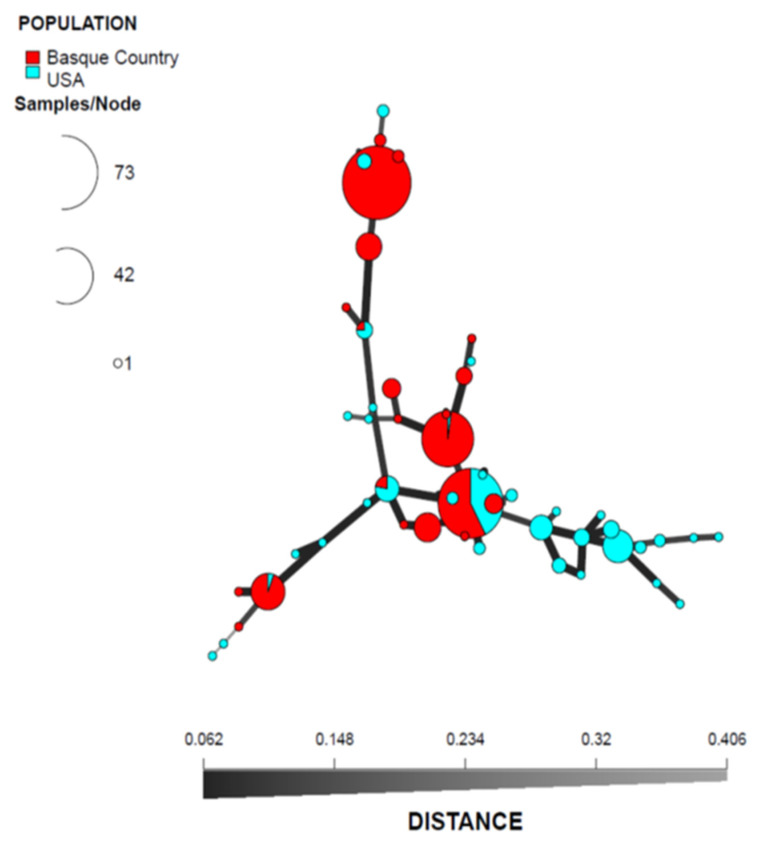
Minimum spanning network from the clone-corrected data showing the relationships among the individual multilocus genotypes (MLGs) found among the populations defined by country of origin (the Basque Country in Spain and USA). Each node represents a different MLG. The distances and thicknesses of the lines between nodes are proportional to Bruvo’s distance [63]. Node colors and sizes correspond to the population studied and the number of individuals, respectively.

All primer pairs evaluated successfully amplified SSR loci for *D. sapinea* from the Basque Country and the USA. Eight loci (SS9, SS10, SS11, SS12, SS13, SS14, SS15 and SS16) were polymorphic for the 322 isolates genotyped. The number of observed alleles per locus ranged from two to nine (Table 1), resulting in a total of 48 MLGs (Table 2). The Basque Country population exhibited 19 MLGs, the USA population exhibited 34 MLGs, and both populations shared five MLGs (Figure 1). A clone correction of the dataset was performed to remove the bias of resampled MLG in the analysis, resulting in a total of 53 representative isolates.

The USA population showed higher genetic diversity (G = 34) than the Basque Country population (G = 19). Similarly, the Shannon-Weiner diversity index (H) for the USA population was higher (3.53) than that observed for the Basque Country population (2.94) (Table 2). When considering the population defined by the nursery location within the Basque Country, Laukiniz and Hernani showed the highest genetic diversity based on values of G (six and eight, respectively), evenness (0.427 and 0.407, respectively) and H (1.79 and 2.08, respectively) (Table 2). Among the populations defined by sample type within the Basque Country, the cone population showed the highest genetic diversity based on the same indices (Table 2). The Basque Country population showed no significant deviation in the rbarD value from the null hypothesis of recombination, supporting sexual reproduction (rbarD = −0.0739; *p* = 0.999).

Pairwise G´st values calculated on the clone-corrected data showed very low genetic differentiation among Basque Country and USA populations (G´st = 0.161; *p* > 0.01). In general, low genetic differentiation was also observed among populations defined by nursery location within the Basque Country or by sample type. G´st values were above 0.04 when the Laukiniz population was compared with Sollano and Oiartzun (0.1303 and 0.0809, respectively). The results of the population subdivision analysis based on G´st were consistent with those obtained by AMOVA. Analysis of molecular variance on the clone-corrected data revealed only 9.5% variation between nursery populations (*p* = 0.024). None of the calculated values were statistically significant, showing that further sampling is likely needed.

**Figure 2:**
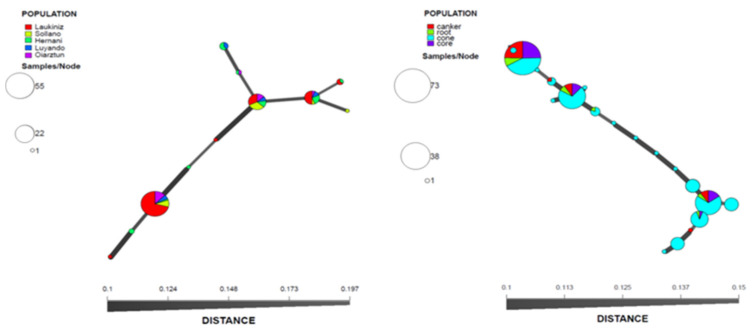
Minimum spanning network from the clone-corrected data showing the relationships among the individual multilocus genotypes (MLGs) found among populations defined by A. geographical location of the nurseries within the Basque Country and B. type of material. Each node represents a different MLG. The distances and thicknesses of the lines between nodes are proportional to Bruvo’s distance [63]. Node colors and sizes correspond to the population studied and the number of individuals, respectively.

In the Basque Country, 11 out of the 19 MLGs identified were present in the populations defined by nursery location, and three MLGs were shared among all populations (Figure 2A). The Sollano population showed one exclusive MLG, while the Laukiniz population showed two exclusive MLGs and one shared with the Hernani population. Luyando and Oiartzun populations also showed one MLG shared with the Hernani population. This last population also showed two exclusive MLGs.

For populations in the Basque Country defined by sample type (canker, root, cone and core), among the 19 MLGs, only three were shared by all populations (Figure 2B). The canker population showed one exclusive MLG and one shared with the cone population. The root population showed an MLG shared with the cone population and an MLG shared with the core population. Finally, the cone population showed 12 exclusive MLGs. Of all the samples analyzed, a higher isolation of *D. sapinea* strains and density of pycnidia were always observed in the samples obtained from cone scales.

### 3.3. DNA Sequencing and Phylogenetic Analysis

Spanish *D. sapinea* isolates belong to one ITS (531 aligned nucleotides) and one TEF1-α (408 aligned nucleotides) haplotype that was equivalent to *D. sapinea* CAA892; Portugal [68].

## 4. Discussion

*Diplodia sapinea* is, by far, the most frequent *Diplodia* species present in *P. radiata* stands in northern Spain. The presence of *D. scrobiculata,* confirmed by morphological and molecular methods, has been reported in only a single tree of all the analyzed samples. This is consistent with what was described by Burgess et al. [69], in which *D. scrobiculata* (formerly known as the B morphotype of *D. pinea*) was considered to have a much more limited distribution in the USA and Mexico, where it was found to coexist with *D. sapinea. D. scrobiculata* has only been reported sporadically in Europe in Mediterranean areas [27,70]. It was only detected on *P. radiata* in Corsica, France [12] and Bizkaia, Spain [31]. Although the distribution of *D. scrobiculata* in Spain detected in this study was extremely limited, the hypothesis that the replacement of this species by the more aggressive *D. sapinea* seems unlikely. Inoculation trials have shown that some of the *D. scrobiculata* isolates were as virulent as those of *D. sapinea* on *P. radiata* and *Pinus elliottii* Engelm [31,52]. Other studies have shown that *D. sapinea* isolates were more aggressive than *D. scrobiculata* isolates to young *P. resinosa, P. banksiana, Pinus sylvestris* L., *Pinus mugo* Turra, *Picea pungens* Engelm, *Pseudotsuga menziesii* (Mirb.) Franco, and *Abies balsamea* (L.) Mil. trees in a greenhouse setting [33,71]. 

*Diplodia sapinea* is also the most frequently encountered *Diplodia* species in samples collected from *P. resinosa* in the northeastern USA. This result is consistent with predominance of *D. sapinea* in North America. For example, in previous studies in Wisconsin, most cones and asymptomatic shoots from which *Diplodia* species were identified, were positive for *D. sapinea,* with only <13% of *P. resinosa* cones collected from the canopy and asymptomatic shoots were positive for *D. scrobiculata* [50,72,73]. Red pine cones collected in New England for the current study yielded only *D. sapinea* isolates. Both studied *Diplodia* species may have a preference in host range, as *D. scrobiculata* is more frequently isolated from *P. banksiana* than *P. resinosa,* whereas *D. sapinea* is more frequently isolated from *P. resinosa* than from *P. banksiana* cones from mature trees [72].

Expansion of the know range of *D. sapinea* to the northern regions in Europe is well documented. It has recently been detected in relatively cold areas such as Estonia [74], Sweden [75], Finland [76] and northwestern Russia [77]. In the current study, *D. sapinea* was isolated from wood, roots and mainly from cones of *P. radiata,* in which the fruiting bodies of this species are found more easily and abundantly than in the rest of the tree parts. The fungus is commonly found as a saprophyte in cone bracts [78,79,80]. In Finland, the frequency of cones with pycnidia of *D. sapinea* varied from 1% to 12% in the infested *P. sylvestris* stands [76]. No symptoms of Diplodia tip blight or resinous cankers were detected in trees in the Finnish stands where the cones were collected. Since fruiting bodies on cones are easily seen and cones can be collected from the soil without having to climb the tree, it is an effective source of fungal material. In some studies, the frequency of cone colonization by *D. sapinea* was considered a measure of the level of pathogen presence in the stands [12,50]. However, this type of extrapolation is susceptible to errors derived from a sampling bias [81].

The genetic diversity, population structure and mode of reproduction of *D. sapinea* were assessed using previously developed microsatellite markers. Analyzed populations were defined based on the country of origin (the Basque Country in Spain and USA), sampling intensity stands within the Basque Country and sample type. The population from the USA showed a higher number of genotypes compared to those in Spain, as expected from a well-established pathogen in a country, taking into account that USA isolates came from a wider area and different pine species. However, the genetic diversity observed in the Basque Country was high relative to the low variability found in previous studies in Spain [82]. An important difference between the previous and the present study is the sampling that was intensively performed within Basque Country plantations. Nevertheless, this result contrasts with other studies of the fungus showing that genetic diversity among *D. sapinea* populations is low at the global scale [27,83,84]. This contradiction may be due to methodological differences. The low genetic diversity has been explained based on the success of some genotypes as endophytes. The close association of *D. pinea* as an endophyte with pines suggests the ability to overcome pressure regardless of the external environmental conditions [84]. This fact coupled with no evidence of sexual reproduction would explain the clonality found within global populations of *D. pinea*, although recent studies showed a cryptic sexual stage in this species [82]. 

Regarding the pathogen mode of reproduction in Spain, linkage disequilibrium analysis showed evidence of recombination in the *D. sapinea* population of the Basque Country. This result contrasts with the idea of predominantly asexual reproduction of the pathogen in Europe based on the clonal structure found across the continent [82,83].

When considering the population based on the geographical location of the nurseries within the Basque Country, Laukiniz and Hernani showed the highest genetic diversity based on the estimated parameters. Among intensively sampled locations, the highest diversity was expected to be detected in Laukiniz since this location was a nursery that received *P. radiata* material, mainly seeds, from different countries and distributed the material through different surrounding locations. This fact is supported by the population subdivision results showing an overall lack of structure based on geographic location within the Basque Country. However, measures of genetic differentiation showed higher values in pairwise comparisons between Laukiniz and Sollano and Laukiniz and Oiartzun. Subdivision among Laukiniz and other populations within the Basque Country might be explained by the fact that the main sources of *P. radiata* seeds in this location were imported from countries such as the USA, Chile and New Zealand, and seeds are a common disease propagation system for this species.

Among the populations defined by sample type within the Basque Country, the cone population showed the highest genetic diversity based on the estimated parameters. These results are in accordance with the success and stability of *D. sapinea* as an endophyte in healthy trees [51]. From the results obtained in this study, it can be inferred that the plant material should be considered for the sampling strategy. Sampling carried out based on exclusively cones could give problems of biasing the results since the population structure may differ depending on the type of sample. Despite the fact that in certain situations, global scaling could potentially provide better results than local scaling [85], this study once again emphasizes the importance of enhancing hierarchical studies, including intensive sampling at the local scale and matching scales of disease monitoring with scales of management systems, avoiding scale discordance. Forest managers are affected by forest policies formulated at the subnational, national, regional and global levels. National climate change policies are influenced by global factors and regional policies but adapted to local circumstances [FAO, http://www.fao.org/3/i3383e/i3383e.pdf, accessed on 10 October 2021]. Forest managers should be aware of forest ecosystem issues at global and local scales that will affect them directly or indirectly, such as the disease impact, epidemiology and genetic diversity of *D. sapinea,* which will lead to disease outbreaks when trees are physiologically stressed [52]. Disease management should focus on reducing inoculum pressure (inoculum present in pruning and felling remains, pine cones, etc. colonized by *D. sapinea*) and new sources of entry of genetic diversity of the pathogen.

Pathogens impose strong selective pressures on their hosts. The way in which fungal populations respond to adverse conditions, such as those generated by control strategies, determines the risk of management failure due to pathogen adaptation conditioned by the type of genetic variability available.

Changes in weather and climate may have implications for development of Diplodia tip blight. In northern Spain, spring temperatures have shown an increase over recent decades [86]. According to the Intergovernmental Panel on Climate Change (IPCC), climate change could increase average temperatures by 2–4 °C in Europe over the next 50 years and cause considerable changes in regional and seasonal patterns of precipitation. This will alter the environmental conditions to which forest trees in Europe are adapted and expose them to new and old diseases. The increased temperature could also increase trees’ susceptibility to disease due to increased exposure to drought. Generation of disease databases at local and global scales in regional climate change scenarios is one of the fundamental starting points in assessing impacts, vulnerability and future needs with respect to adaptation to Diplodia tip blight forest outbreaks [16,44,82,87].

## Figures and Tables

**Table 1:** *Diplodia sapinea* isolates used in this study: origin, host species, sample type and strain identification code.

Origin	Host Species	Sample Type	Sample ID	Origin	Host Species	Sample Type	Sample ID
Bajo Deba, Spain	*Pinus pinaster*	Canker	BCI2	P1 (Laukiniz), Spain	*Pinus radiata*	Canker	BCM198
Plentzia-Munguia, Spain	*Pinus radiata*	Root	BCI5	P1 (Laukiniz), Spain	*Pinus radiata*	Canker	BCM199
Alto Deba, Spain	*Pinus radiata*	Cone	BC14	P1 (Laukiniz), Spain	*Pinus radiata*	Canker	BCM200
Estribaciones del Gorbea, Spain	*Pinus radiata*	Cone	BC15	P1 (Laukiniz), Spain	*Pinus radiata*	Canker	BCM201
Encartaciones, Spain	*Pinus radiata*	Cone	BC17	P1 (Laukiniz), Spain	*Pinus radiata*	Canker	BCM202
Encartaciones, Spain	*Pinus radiata*	Cone	BC18	P2 (Sollano), Spain	*Pinus radiata*	Cone	BC22
Encartaciones, Spain	*Pinus radiata*	Cone	BC19	P2 (Sollano), Spain	*Pinus radiata*	Cone	BC23
Alto Deba, Spain	*Pinus radiata*	Cone	BC39	P2 (Sollano), Spain	*Pinus radiata*	Cone	BC24
Alto Deba, Spain	*Pinus radiata*	Cone	BC40	P2 (Sollano), Spain	*Pinus radiata*	Cone	BC25
Goierri, Spain	*Pinus nigra*	Cone	BC41	P2 (Sollano), Spain	*Pinus radiata*	Cone	BC26
Goierri, Spain	*Pinus nigra*	Cone	BC42	P2 (Sollano), Spain	*Pinus radiata*	Cone	BC27
Montaña Alavesa, Spain	*Pinus nigra*	Cone	BC44	P2 (Sollano), Spain	*Pinus radiata*	Cone	BC29
Llanada Alavesa, Spain	*Pinus radiata*	Cone	BC45	P2 (Sollano), Spain	*Pinus radiata*	Cone	BC30
Llanada Alavesa, Spain	*Pinus radiata*	Cone	BC46	P2 (Sollano), Spain	*Pinus radiata*	Cone	BC31
Llanada Alavesa, Spain	*Pinus radiata*	Cone	BC47	P2 (Sollano), Spain	*Pinus radiata*	Cone	BC33
Gernika Bermeo, Spain	*Pinus radiata*	Cone	BC48	P2 (Sollano), Spain	*Pinus radiata*	Cone	BC34
Gernika Bermeo, Spain	*Pinus radiata*	Cone	BC49	P2 (Sollano), Spain	*Pinus radiata*	Cone	BC35
Gernika Bermeo, Spain	*Pinus radiata*	Cone	BC50	P2 (Sollano), Spain	*Pinus radiata*	Cone	BC36
Gernika Bermeo, Spain	*Pinus radiata*	Cone	BC51	P2 (Sollano), Spain	*Pinus radiata*	Cone	BC38
Gernika Bermeo, Spain	*Pinus radiata*	Cone	BC52	P3 (Hernani), Spain	*Pinus radiata*	Cone	BC60
Duranguesado, Spain	*Pinus radiata*	Cone	BC53	P3 (Hernani), Spain	*Pinus radiata*	Cone	BC61
Markina-Ondarroa, Spain	*Pinus radiata*	Cone	BC54	P3 (Hernani), Spain	*Pinus radiata*	Cone	BC62
Donostia San Sebastian, Spain	*Pinus radiata*	Cone	BC55	P3 (Hernani), Spain	*Pinus radiata*	Cone	BC63
Valles Alaveses, Spain	*Pinus attenuata*	Cone	BC56	P3 (Hernani), Spain	*Pinus radiata*	Cone	BC64
Gernika Bermeo, Spain	*Pinus radiata*	Cone	BC57	P3 (Hernani), Spain	*Pinus radiata*	Cone	BC65
Goierri, Spain	*Pinus radiata*	Cone	BC76	P3 (Hernani), Spain	*Pinus radiata*	Cone	BC66
Goierri, Spain	*Pinus radiata*	Cone	BC77	P3 (Hernani), Spain	*Pinus radiata*	Cone	BC67
Goierri, Spain	*Pinus radiata*	Cone	BC78	P3 (Hernani), Spain	*Pinus radiata*	Cone	BC68
Tolosa, Spain	*Pinus radiata*	Cone	BC79	P3 (Hernani), Spain	*Pinus radiata*	Cone	BC69
Tolosa, Spain	*Pinus radiata*	Cone	BC80	P3 (Hernani), Spain	*Pinus radiata*	Cone	BC70
Tolosa, Spain	*Pinus radiata*	Cone	BC81	P3 (Hernani), Spain	*Pinus radiata*	Cone	BC71
Tolosa, Spain	*Pinus nigra*	Cone	BC82	P3 (Hernani), Spain	*Pinus radiata*	Cone	BC72
Tolosa, Spain	*Pinus nigra*	Cone	BC83	P3 (Hernani), Spain	*Pinus radiata*	Cone	BC73
Tolosa, Spain	*Pinus nigra*	Cone	BC84	P3 (Hernani), Spain	*Pinus radiata*	Cone	BC74
Tolosa, Spain	*Pinus radiata*	Cone	BC86	P3 (Hernani), Spain	*Pinus radiata*	Cone	BC75
Donostia San Sebastian, Spain	*Pinus radiata*	Cone	BC87	P4 (Luyando), Spain	*Pinus radiata*	Root	BCM157
Donostia San Sebastian, Spain	*Pinus radiata*	Cone	BC89	P4 (Luyando), Spain	*Pinus radiata*	Root	BCM175
Donostia San Sebastian, Spain	*Pinus radiata*	Cone	BC90	P4 (Luyando), Spain	*Pinus radiata*	Root	BCM176
Donostia San Sebastian, Spain	*Pinus radiata*	Cone	BC91	P4 (Luyando), Spain	*Pinus radiata*	Root	BCM177
Donostia San Sebastian, Spain	*Pinus radiata*	Cone	BC92	P4 (Luyando), Spain	*Pinus radiata*	Root	BCM178
Donostia San Sebastian, Spain	*Pinus radiata*	Cone	BC93	P4 (Luyando), Spain	*Pinus radiata*	Core	BCM179
Urola Costa, Spain	*Pinus radiata*	Cone	BC94	P4 (Luyando), Spain	*Pinus radiata*	Root	BCM181
Urola Costa, Spain	*Pinus radiata*	Cone	BC95	P4 (Luyando), Spain	*Pinus radiata*	Root	BCM183
Urola Costa, Spain	*Pinus radiata*	Cone	BC96	P4 (Luyando), Spain	*Pinus radiata*	Core	BCM187
Urola Costa, Spain	*Pinus radiata*	Cone	BC97	P5 (Oiarztun), Spain	*Pinus radiata*	Core	BCM158
Bajo Deba, Spain	*Pinus radiata*	Cone	BC98	P5 (Oiarztun), Spain	*Pinus radiata*	Core	BCM159
Bajo Deba, Spain	*Pinus radiata*	Cone	BC99	P5 (Oiarztun), Spain	*Pinus radiata*	Core	BCM160
Encartaciones, Spain	*Pinus radiata*	Cone	BC100	P5 (Oiarztun), Spain	*Pinus radiata*	Core	BCM162
Encartaciones, Spain	*Pinus radiata*	Cone	BC101	P5 (Oiarztun), Spain	*Pinus radiata*	Core	BCM163
Encartaciones, Spain	*Pinus nigra*	Cone	BC104	P5 (Oiarztun), Spain	*Pinus radiata*	Core	BCM164
Encartaciones, Spain	*Pinus radiata*	Cone	BC105	P5 (Oiarztun), Spain	*Pinus radiata*	Core	BCM173
Encartaciones, Spain	*Pinus radiata*	Cone	BC106	P5 (Oiarztun), Spain	*Pinus radiata*	Core	BCM180
Encartaciones, Spain	*Pinus radiata*	Cone	BC108	P5 (Oiarztun), Spain	*Pinus radiata*	Root	BCM209
Valles Alaveses, Spain	*Pinus pinaster*	Cone	BC109	P5 (Oiarztun), Spain	*Pinus radiata*	Root	BCM211
Estribaciones del Gorbea, Spain	*Pinus radiata*	Cone	BC112	P5 (Oiarztun), Spain	*Pinus radiata*	Root	BCM212
Arratia Nervión, Spain	*Pinus nigra*	Cone	BC113	P5 (Oiarztun), Spain	*Pinus radiata*	Root	BCM213
Arratia Nervión, Spain	*Pinus radiata*	Cone	BC114	Barbour County, West Virginia USA	*Pinus sylvestris*	Needle	W171
Cantábrica Alavesa, Spain	*Pinus radiata*	Cone	BC115	Black Hills, South Dakota USA	*Pinus ponderosa*	Stem	W172
Cantábrica Alavesa, Spain	*Pinus radiata*	Cone	BC116	Idaho USA	*Pinus ponderosa*	Unknown	W174
Valles Alaveses, Spain	*Pinus halepensis*	Cone	BC120	Grant County, Wisconsin USA	*Pinus resinosa*	Unknown	W175
Valles Alaveses, Spain	*Pinus sylvestris*	Cone	BC121	Oktibbeha County, Mississippi USA	*Pinus palustris*	Unknown	W177
Valles Alaveses, Spain	*Pinus contorta*	Cone	BC123	Pennsylvania USA	*Pinus sylvestris*	Unknown	W180
Estribaciones del Gorbea, Spain	*Pinus radiata*	Cone	BC124	Riverside County, California USA	*Pinus jeffreyi*	Unknown	W185
Cantábrica Alavesa, Spain	*Pinus radiata*	Cone	BC125	Florida USA	*Pinus elliottii*	Cone-seed	W186
Arratia Nervión, Spain	*Pinus radiata*	Cone	BC127	Bennington County, Vermont USA	*Pinus resinosa*	Cone	W190
Llanada Alavesa, Spain	*Pinus radiata*	Cone	BC128	Maui, Hawaii USA	*Pinus radiata*	Unknown	W191
Llanada Alavesa, Spain	*Pinus radiata*	Cone	BC129	Itasca St. Park, Minnesota USA	*Pinus resinosa*	Unknown	W192
Spain	*Pinus nigra*	Cone	BC131	Dallas County, Texas USA	*Pinus eldarica*	Cone	W194
Llanada Alavesa, Spain	*Pinus nigra*	Cone	BC132	Georgia USA	*Pinus taeda*	Needle	W206
Llanada Alavesa, Spain	*Pinus radiata*	Cone	BC133	Wisconsin USA	*Pinus banksiana*	Needle	W210
Llanada Alavesa, Spain	*Pinus radiata*	Cone	BC134	Wisconsin USA	*Pinus banksiana*	Needle	W211
Llanada Alavesa, Spain	*Pinus radiata*	Cone	BC135	Waushara County, Wisconsin USA	*Pinus resinosa*	Needle	W212
Llanada Alavesa, Spain	*Pinus radiata*	Cone	BC137	Portage County, Wisconsin USA	*Pinus banksiana*	Needle	W213
Llanada Alavesa, Spain	*Pinus radiata*	Cone	BC138	Portage County, Wisconsin USA	*Pinus resinosa*	Needle	W214
Montaña Alavesa, Spain	*Pinus radiata*	Cone	BC141	Wood County, Wisconsin USA	*Pinus resinosa*	Needle	W215
Alto Deba, Spain	*Pinus radiata*	Cone	BC142	Adams County, Wisconsin USA	*Pinus resinosa*	Needle	W216
Bajo Deba, Spain	*Pinus radiata*	Cone	BC143	Marathon County, Wisconsin USA	*Pinus resinosa*	Needle	W217
Bajo Deba, Spain	*Pinus radiata*	Cone	BC144	Wallowa County, Oregon USA	*Pinus ponderosa*	Cone	W218
Bajo Deba, Spain	*Pinus radiata*	Cone	BC145	Wallowa County, Oregon, USA	*Pinus ponderosa*	Needle	W219
Markina Ondarroa, Spain	*Pinus radiata*	Cone	BC146	Bennington County, Vermont USA	*Pinus resinosa*	Stem tip	W220
Estribaciones del Gorbea, Spain	*Pinus radiata*	Cone	BC147	Sawyer County, Wisconsin USA	*Pinus banksiana*	Stem	W221
Arratia Nervión, Spain	*Pinus radiata*	Cone	BC148	Wood County, Wisconsin USA	*Pinus banksiana*	Stem	W222
Arratia Nervión, Spain	*Pinus radiata*	Cone	BC149	Vilas County, Wisconsin USA	*Pinus resinosa*	Unknown	W223
Encartaciones, Spain	*Pinus radiata*	Cone	BC150	South Dakota	*Pinus ponderosa*	Unknown	W224
Encartaciones, Spain	*Pinus radiata*	Cone	BC151	Dallas County, Texas USA	*Pinus nigra*	Cone	W225
Encartaciones, Spain	*Pinus radiata*	Cone	BC152	Bayfield County, Wisconsin USA	*Pinus resinosa*	Needle	W226
Duranguesado, Spain	*Pinus radiata*	Cone	BC154	Sumter County, Alabama USA	*Pinus taeda*	Cone	W227
Gernika Bermeo, Spain	*Pinus radiata*	Cone	BC155	Adams County, Wisconsin USA	*Pinus sylvestris*	Bark	W228
Gernika Bermeo, Spain	*Pinus radiata*	Cone	BC156	Vilas County, Wisconsin USA	*Pinus ponderosa*	Unknown	W229
Markina-Ondarroa, Spain	*Pinus radiata*	Cone	BC157	Vilas County, Wisconsin USA	*Pinus resinosa*	Unknown	W231
Markina-Ondarroa, Spain	*Pinus radiata*	Cone	BC158	Pine County, Minnesota USA	*Pinus resinosa*	Unknown	W232
Duranguesado, Spain	*Pinus radiata*	Cone	BC159	Morgantown, WV	*Pinus nigra*	Needle	W233
Gernika Bermeo, Spain	*Pinus radiata*	Cone	BC160	Jackson County, Wisconsin USA	*Pinus resinosa*	Needle	W234
Spain	*Pinus radiata*	Cone	BC161	Dane County, Wisconsin USA	*Pinus nigra*	Needle	W235
Plentzia-Munguia, Spain	*Pinus radiata*	Cone	BC162	Northern Highland American Legion State Forest, Wisconsin USA	*Pinus banksiana*	Twig	W236
Goierri, Spain	*Pinus radiata*	Cone	BC163	Marquette County, Wisconsin USA	*Pinus resinosa*	Needle	W238
Tolosa, Spain	*Pinus radiata*	Cone	BC164	Trempealeau County, Wisconsin USA	*Pinus resinosa*	Needle	W239
Tolosa, Spain	*Pinus radiata*	Cone	BC165	Fairfield County, Connecticut USA	*Pinus sylvestris*	Needle	W240
Urola Costa, Spain	*Pinus radiata*	Cone	BC166	Adair County, Iowa USA	*Pinus nigra*	Needle	W241
Markina-Ondarroa, Spain	*Pinus radiata*	Cone	BC169	Codington County, South Dakota USA	*Pinus sylvestris*	Needle	W242
Cantábrica Alavesa, Spain	*Pinus radiata*	Cone	BC196	Polk County, Iowa USA	*Pinus nigra*	Needle	W243
Arratia Nervión, Spain	*Pinus radiata*	Cone	BC197	Lacrosse County, Wisconsin USA	*Pinus resinosa*	Needle	W244
Arratia Nervión, Spain	*Pinus radiata*	Cone	BC198	Wood County, Wisconsin USA	*Pinus resinosa*	Stem	W245
Arratia Nervión, Spain	*Pinus radiata*	Cone	BC199	Centre County, Pennsylvania USA	*Pinus nigra*	Needle	W246
Arratia Nervión, Spain	*Pinus radiata*	Cone	BC200	Upshur County, West Virginia USA	*Pinus sylvestris*	Needle	W247
Duranguesado, Spain	*Pinus radiata*	Cone	BC201	Lafayette County, Wisconsin USA	*Pinus resinosa*	Needle	W248
Gran Bilbao, Spain	*Pinus radiata*	Cone	BC202	Monroe County, Wisconsin USA	*Pinus banksiana*	Needle	W250
Plentzia-Munguia, Spain	*Pinus radiata*	Cone	BC203	Cheboygan County, Michigan USA	*Pinus banksiana*	Needle	W251
Plentzia-Munguia, Spain	*Pinus radiata*	Stem	BC208	Manistee National Forest, Michigan USA	*Pinus resinosa*	Unknown	W252
Plentzia-Munguia, Spain	*Pinus radiata*	Stem	BC209	Jefferson County, West Virginia USA	*Pinus nigra*	Needle	W253
P1 (Laukiniz), Spain	*Pinus radiata*	Cone	BC2	Stanislaus National Forest, California USA	*Pinus ponderosa*	Unknown	W254
P1 (Laukiniz), Spain	*Pinus radiata*	Cone	BC3	Morgan County, West Virginia USA	*Pinus sylvestris*	Needle	W255
P1 (Laukiniz), Spain	*Pinus radiata*	Cone	BC4	Marion County, Indiana USA	*Pinus nigra*	Cone	W256
P1 (Laukiniz), Spain	*Pinus radiata*	Cone	BC5	Foxborough, Massachusetts, USA	*Pinus resinosa*	Cone	U1
P1 (Laukiniz), Spain	*Pinus radiata*	Cone	BC6	Washington, Vermont, USA	*Pinus resinosa*	Cone	U2
P1 (Laukiniz), Spain	*Pinus radiata*	Cone	BC7	Hancock County, Maine, USA	*Pinus resinosa (natural*)	Cone	U3
P1 (Laukiniz), Spain	*Pinus radiata*	Cone	BC8	Hancock County, Maine, USA	*Pinus resinosa (natural*)	Cone	U4
P1 (Laukiniz), Spain	*Pinus radiata*	Cone	BC9	Andover, Massachusetts, USA	*Pinus resinosa*	Cone	U5
P1 (Laukiniz), Spain	*Pinus radiata*	Cone	BC10	Hancock County, Maine, USA	*Pinus resinosa (natural*)	Cone	U6
P1 (Laukiniz), Spain	*Pinus radiata*	Cone	BC11	Washington, Vermont, USA	*Pinus resinosa*	Cone	U7
P1 (Laukiniz), Spain	*Pinus radiata*	Core	BCI1	Washington, Vermont, USA	*Pinus resinosa*	Cone	U8
P1 (Laukiniz), Spain	*Pinus radiata*	Core	BCI3	Andover, Massachusetts, USA	*Pinus resinosa*	Cone	U9
P1 (Laukiniz), Spain	*Pinus radiata*	Core	BCI4	Foxborough, Massachusetts, USA	*Pinus resinosa*	Cone	U10
P1 (Laukiniz), Spain	*Pinus radiata*	Core	BCI6	Shrewsbury, Vermont, USA	*Pinus resinosa*	Cone	U11
P1 (Laukiniz), Spain	*Pinus radiata*	Core	BCI7	Hancock County, Maine, USA	*Pinus resinosa* (natural)	Cone	U12
P1 (Laukiniz), Spain	*Pinus radiata*	Core	BCI8	Shrewsbury, Vermont, USA	*Pinus resinosa*	Cone	U13
P1 (Laukiniz), Spain	*Pinus radiata*	Core	BCI9	Hancock County, Maine, USA	*Pinus resinosa* (natural)	Cone	U14
P1 (Laukiniz), Spain	*Pinus radiata*	Core	BCI10	Hudson, Massachusetts, USA	*Pinus resinosa*	Cone	U15
P1 (Laukiniz), Spain	*Pinus radiata*	Core	BCI11	Shrewsbury, Vermont, USA	*Pinus resinosa*	Cone	U16
P1 (Laukiniz), Spain	*Pinus radiata*	Core	BCI12	Hancock County, Maine, USA	*Pinus resinosa* (natural)	Cone	U17
P1 (Laukiniz), Spain	*Pinus radiata*	Core	BCI13	Hancock County, Maine, USA	*Pinus resinosa* (natural)	Cone	U18
P1 (Laukiniz), Spain	*Pinus radiata*	Core	BCI14	Hancock County, Maine, USA	*Pinus resinosa* (natural)	Cone	U19
P1 (Laukiniz), Spain	*Pinus radiata*	Core	BCI15	Shrewsbury, Vermont, USA	*Pinus resinosa*	Cone	U20
P1 (Laukiniz), Spain	*Pinus radiata*	Core	BCI16	Hudson, Massachusetts, USA	*Pinus resinosa*	Cone	U21
P1 (Laukiniz), Spain	*Pinus radiata*	Core	BCI17	Washington, Vermont, USA	*Pinus resinosa*	Cone	U22
P1 (Laukiniz), Spain	*Pinus radiata*	Core	BCI18	Foxborough, Massachusetts, USA	*Pinus resinosa*	Cone	U23
P1 (Laukiniz), Spain	*Pinus radiata*	Core	BCI19	Hudson, Massachusetts, USA	*Pinus resinosa*	Cone	U24
P1 (Laukiniz), Spain	*Pinus radiata*	Core	BCI20	Hudson, Massachusetts, USA	*Pinus resinosa*	Cone	U25
P1 (Laukiniz), Spain	*Pinus radiata*	Core	BCI21	Washington, Vermont, USA	*Pinus resinosa*	Cone	U26
P1 (Laukiniz), Spain	*Pinus radiata*	Core	BCI22	Andover, Massachusetts, USA	*Pinus resinosa*	Cone	U27
P1 (Laukiniz), Spain	*Pinus radiata*	Canker	BCM161	Washington, Vermont, USA	*Pinus resinosa*	Cone	U28
P1 (Laukiniz), Spain	*Pinus radiata*	Canker	BCM165	Foxborough, Massachusetts, USA	*Pinus resinosa*	Cone	U29
P1 (Laukiniz), Spain	*Pinus radiata*	Canker	BCM167	Washington, Vermont, USA	*Pinus resinosa*	Cone	U30
P1 (Laukiniz), Spain	*Pinus radiata*	Canker	BCM168	Hancock County, Maine, USA	*Pinus resinosa* (natural)	Cone	U31
P1 (Laukiniz), Spain	*Pinus radiata*	Canker	BCM169	Hudson, Massachusetts, USA	*Pinus resinosa*	Cone	U32
P1 (Laukiniz), Spain	*Pinus radiata*	Canker	BCM170	Hancock County, Maine, USA	*Pinus resinosa* (natural)	Cone	U33
P1 (Laukiniz), Spain	*Pinus radiata*	Canker	BCM171	Andover, Massachusetts, USA	*Pinus resinosa*	Cone	U34
P1 (Laukiniz), Spain	*Pinus radiata*	Canker	BCM172	Hancock County, Maine, USA	*Pinus resinosa* (natural)	Cone	U35
P1 (Laukiniz), Spain	*Pinus radiata*	Canker	BCM174	Andover, Massachusetts, USA	*Pinus resinosa*	Cone	U36
P1 (Laukiniz), Spain	*Pinus radiata*	Root	BCM182	Hancock County, Maine, USA	*Pinus resinosa* (natural)	Cone	U37
P1 (Laukiniz), Spain	*Pinus radiata*	Canker	BCM184	Hancock County, Maine, USA	*Pinus resinosa* (natural)	Cone	U38
P1 (Laukiniz), Spain	*Pinus radiata*	Canker	BCM185	Foxborough, Massachusetts, USA	*Pinus resinosa*	Cone	U39
P1 (Laukiniz), Spain	*Pinus radiata*	Canker	BCM186	Andover, Massachusetts, USA	*Pinus resinosa*	Cone	U40
P1 (Laukiniz), Spain	*Pinus radiata*	Canker	BCM188	Washington, Vermont, USA	*Pinus resinosa*	Cone	U41
P1 (Laukiniz), Spain	*Pinus radiata*	Canker	BCM189	Washington, Vermont, USA	*Pinus resinosa*	Cone	U42
P1 (Laukiniz), Spain	*Pinus radiata*	Canker	BCM190	Hancock County, Maine, USA	*Pinus resinosa* (natural)	Cone	U43
P1 (Laukiniz), Spain	*Pinus radiata*	Canker	BCM191	Hancock County, Maine, USA	*Pinus resinosa* (natural)	Cone	U44
P1 (Laukiniz), Spain	*Pinus radiata*	Canker	BCM192	Washington, Vermont, USA	*Pinus resinosa*	Cone	U45
P1 (Laukiniz), Spain	*Pinus radiata*	Canker	BCM193	Hudson, Massachusetts, USA	*Pinus resinosa*	Cone	U46
P1 (Laukiniz), Spain	*Pinus radiata*	Canker	BCM194	Washington, Vermont, USA	*Pinus resinosa*	Cone	U47
P1 (Laukiniz), Spain	*Pinus radiata*	Canker	BCM195	Washington, Vermont, USA	*Pinus resinosa*	Cone	U48
P1 (Laukiniz), Spain	*Pinus radiata*	Canker	BCM196	Shrewsbury, Vermont, USA	*Pinus resinosa*	Cone	U49
P1 (Laukiniz), Spain	*Pinus radiata*	Canker	BCM197	Shrewsbury, Vermont, USA	*Pinus resinosa*	Cone	U50

**Table 2:** Genetic diversity and linkage disequilibrium among loci based on the standardized index of association (rbarD) of *Diplodia sapinea* populations defined by country of origin, by nursery location in the Basque Country and by sample type^a^.

Parameters^b^	Country		Nursery Location in the Basque Country					Sample Type			
	Spain (Basque Country)	USA	Laukiniz	Sollano	Hernani	Luyando	Oiarztun	Canker	Root	Cone	Core
Sample size (N)^c^	216	106	58	14	16	9	12	28	13	145	30
MLG/Diversity (G)	19	34	6	4	8	4	4	5	5	19	4
eMLG	19	19	6	4	8	4	4	5	5	10	4
Evenness (*E*_5_)	1	1	0.427	0.3	0.407	0.333	0.3	1	1	1	1
Diversity (H)	2.94	3.53	1.79	1.39	2.08	1.39	1.39	1.61	1.61	2.94	1.39
rbarD	−0.074	0.087	−0.080	−0.316	−0.066	−0.115	−0.333	−0.056	−0.194	−0.074	−0.115
*p*-value	0.999	0.001									

^a^ The set of nonredundant indices of genotypic diversity recommended by Arnaud-Haond et al. [66] was calculated for each population clone-corrected dataset. ^b^ MLG, number of multilocus genotypes observed; G, Stoddart and Taylor’s diversity [55] genotypic diversity; eMLG, expected multilocus genotypes based on rarefaction; E_5_, evenness index adapted from Simpson diversity; H, Shannon-Weiner diversity index [67]; rbarD, standardized index of association; *p*-value for rbarD. ^c^ Sample size before clone correction.

## Data Availability

Not applicable.
